# Supplementary figures and images for: Modeled larval connectivity of a multi-species reef fish and invertebrate assemblage off the coast of Moloka‘i, Hawai‘i
Source: PeerJ. 2018 Sep 28;6:e5688. doi: 10.7717/peerj.5688 (PMC6166622; doi:10.7717/peerj.5688)

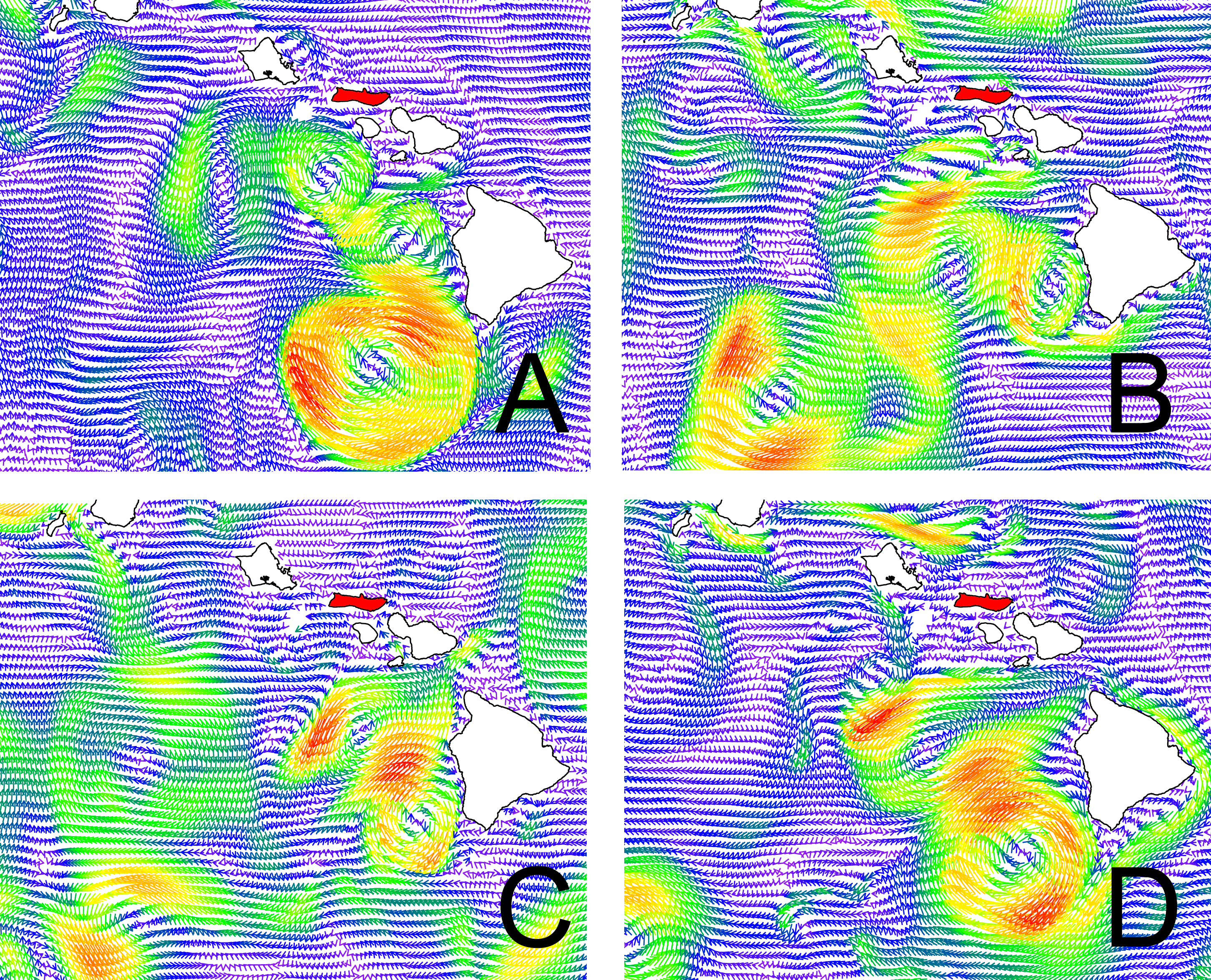

Supplement: Figure S1 — Current direction and velocity is displayed at a depth of 55 m below sea surface on (A) March 31st, 2011, (B) June 30th, 2011, (C) September 30th, 2011, and (D) December 31st, 2011. Arrowhead direction follows current direction, and u/v velocity is displayed through arrow length and color (purple, low velocity, red, high velocity). Domain extends from 198.2°E to 206°E and from 17°N to 22.2°N. The island of Moloka‘i is highlighted in red. [file peerj-06-5688-s001.png]

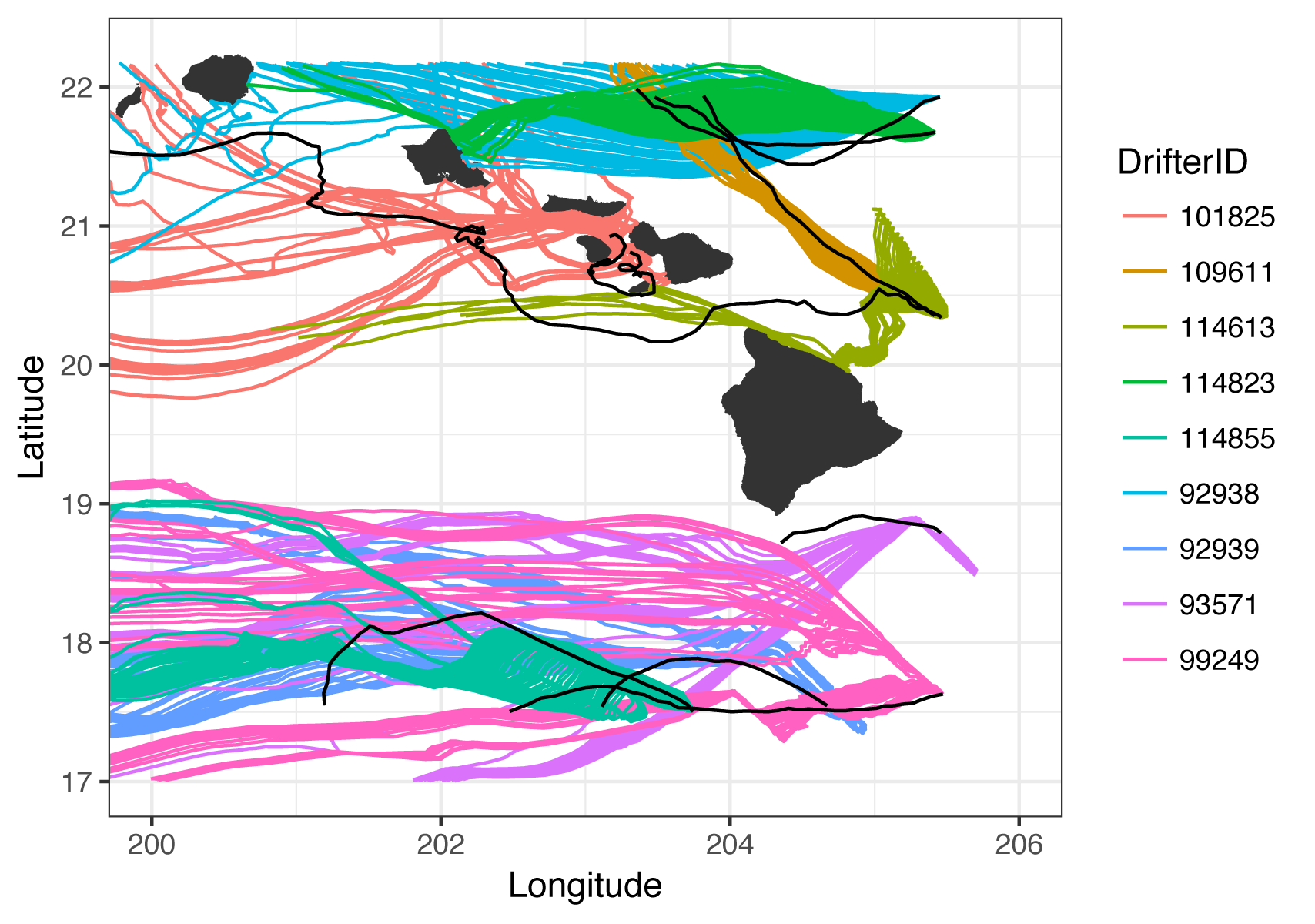

Supplement: Figure S2 — Drifter paths in black and corresponding model paths are colored by drifter ID. All drifter information was extracted from the GDP Drifter Data Assembly Center (Elipot et al., 2016). Drifters were included if they fell within the model domain spatially and temporally, and were tested by releasing 1,000 particles on the correct day where they entered the model domain, at the uppermost depth layer of our oceanographic model (0–5 m). [file peerj-06-5688-s002.png]

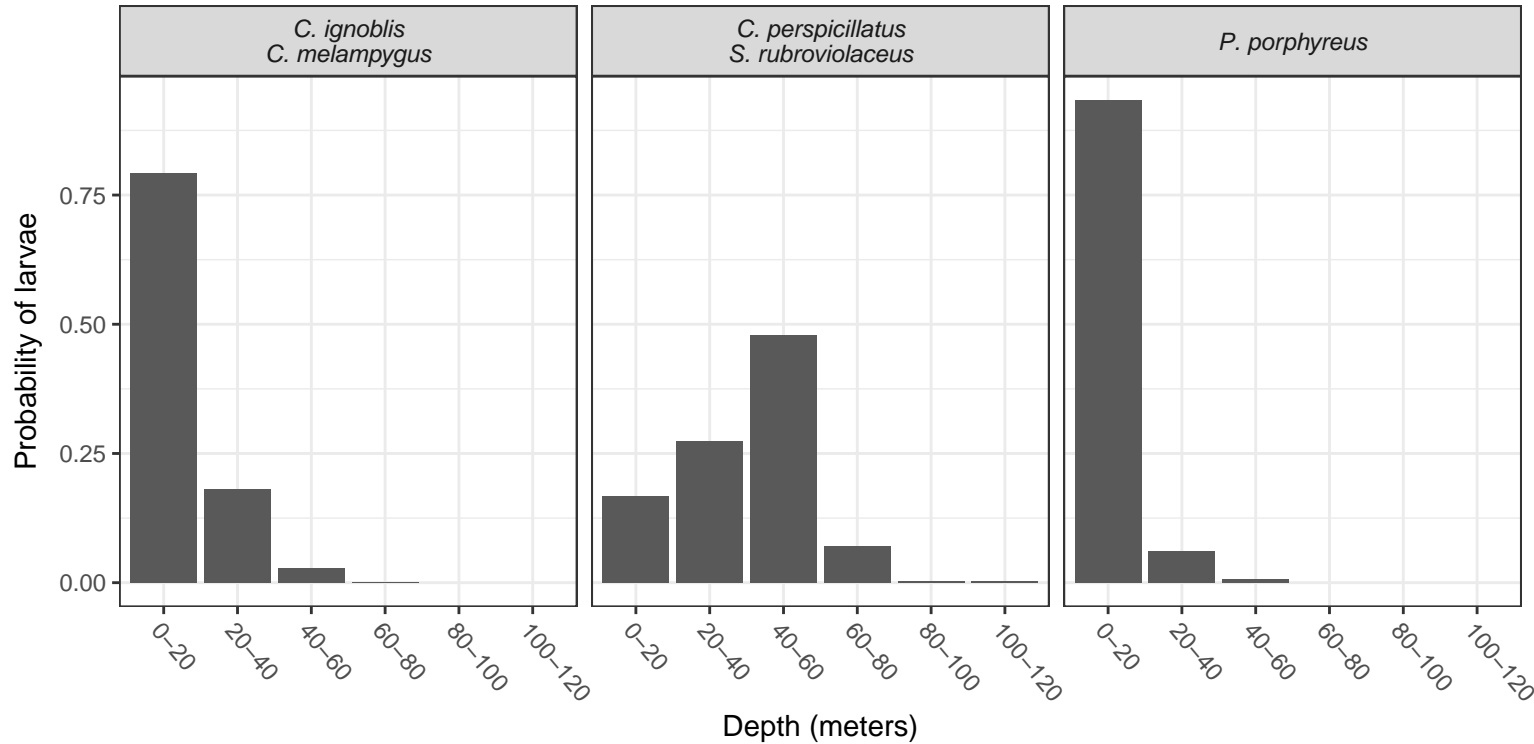

Supplement: Figure S3 — Modeled vertical larval distributions for Caranx spp. (left), S. rubroviolaceus and C. perspicillatus (middle), and P. porphyreus (right), using data from the 1996 NOAA ichthyoplankton vertical distributions data report (Boehlert & Mundy 1996). [file peerj-06-5688-s003.pdf]

Average proportion larvae settled

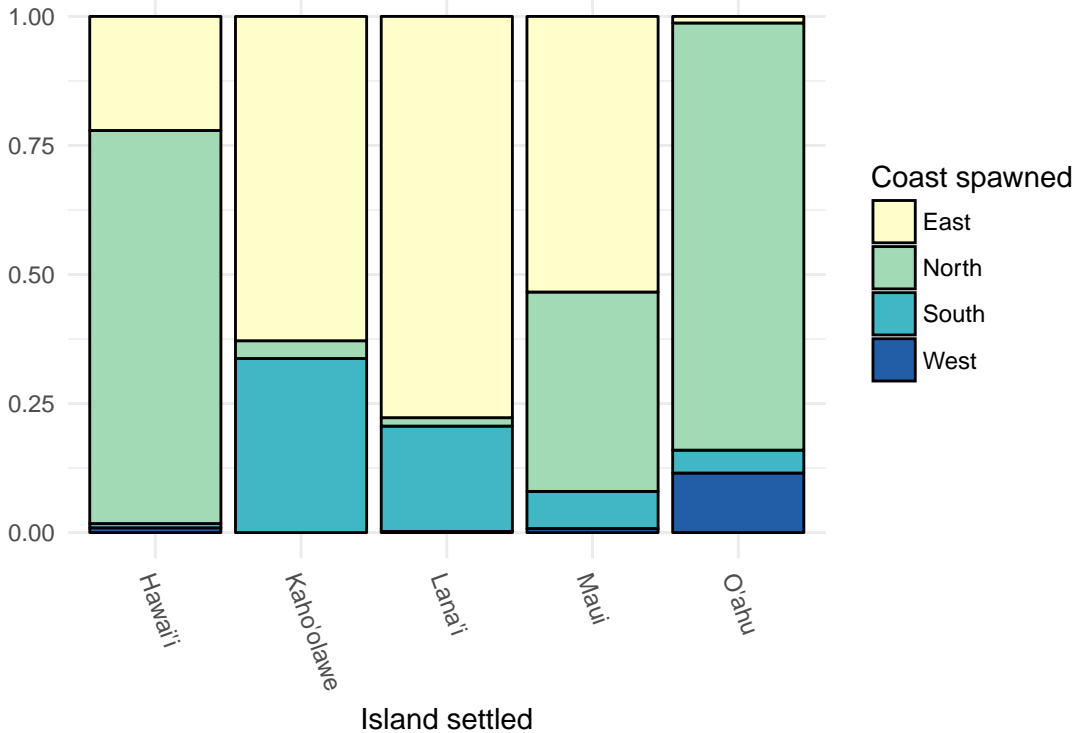

Supplement: Figure S4 — Proportion of simulated larvae settled to each island from sites on each coast of Moloka‘i, averaged across all species that successfully settled to that island. [file peerj-06-5688-s004.pdf]

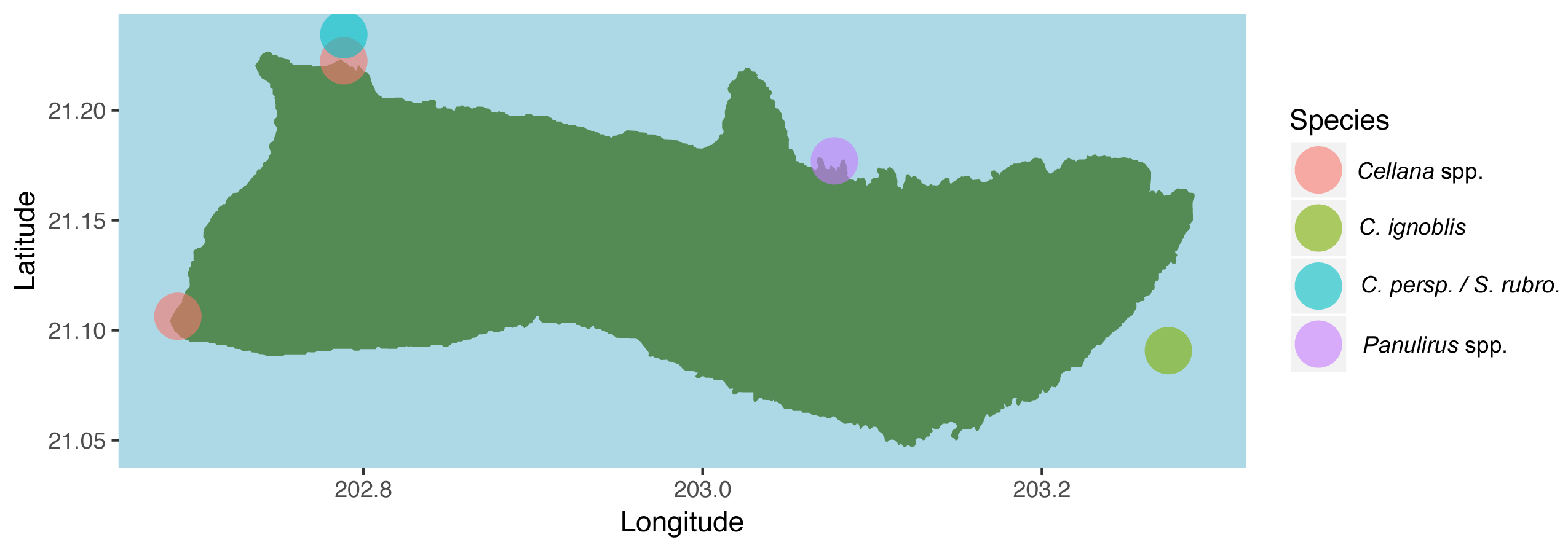

Supplement: Figure S5 — Mokio Point and La‘au Point were cut-nodes for Cellana spp., Mokio Point was a cut-node for C. perspicillatus and S. rubroviolaceus, Pauwalu Harbor and Pala‘au were cut-nodes for C. ignoblis, and Leinaopapio Point was a cut-node for Panulirus spp. [file peerj-06-5688-s005.png]

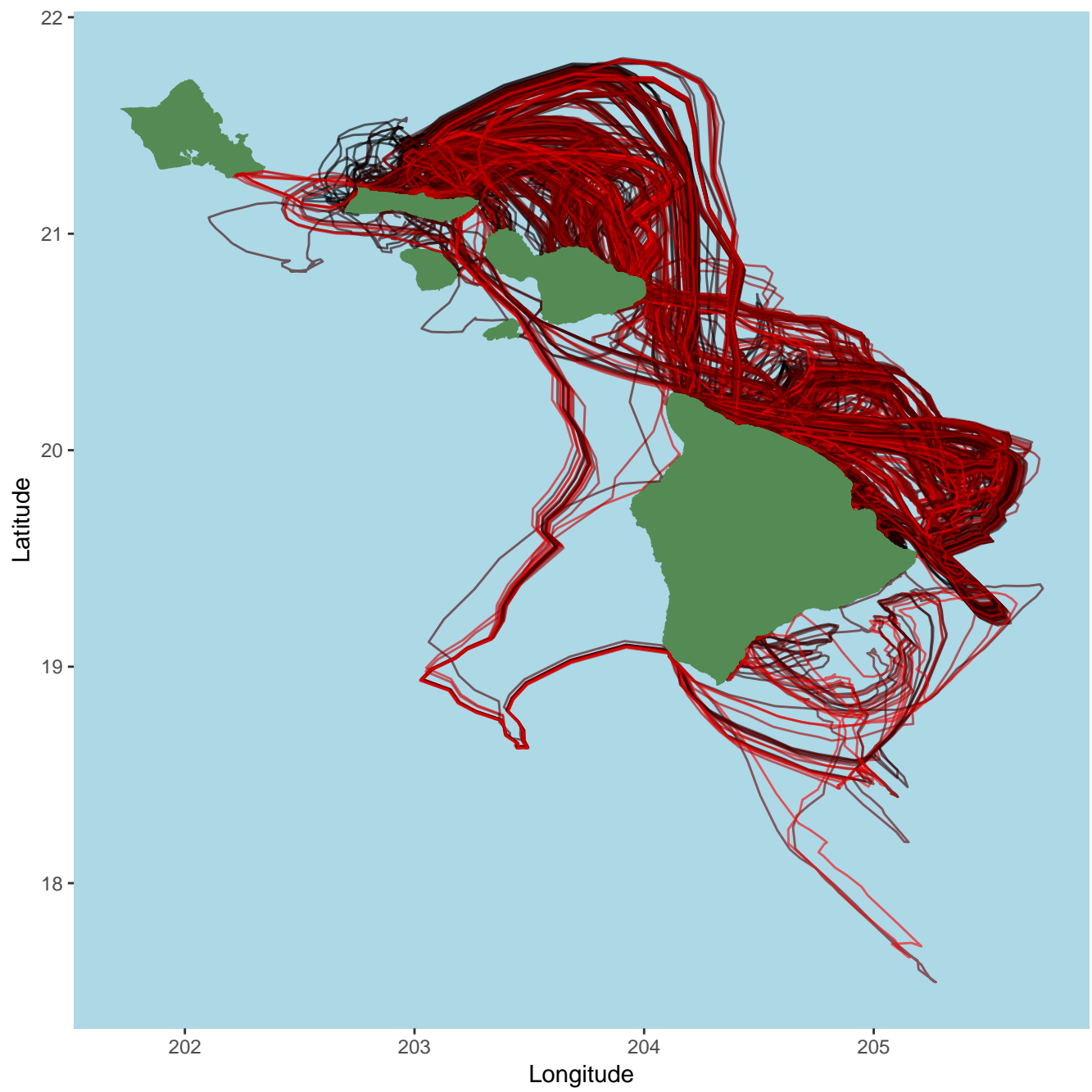

Supplement: Figure S6 — 500 randomly sampled dispersal pathways for lobster larvae (Panulirus spp.) that successfully settled to Hawai‘i Island after being spawned off the coast of Moloka‘i. Red tracks indicate settlement earlier in the year (February–March), while black tracks indicate settlement later in the year (April–May). Most larvae are transported to the northeast coast of Hawai‘i via a gyre to the north of Maui, while a smaller proportion are transported through Maui Nui. [file peerj-06-5688-s006.pdf]

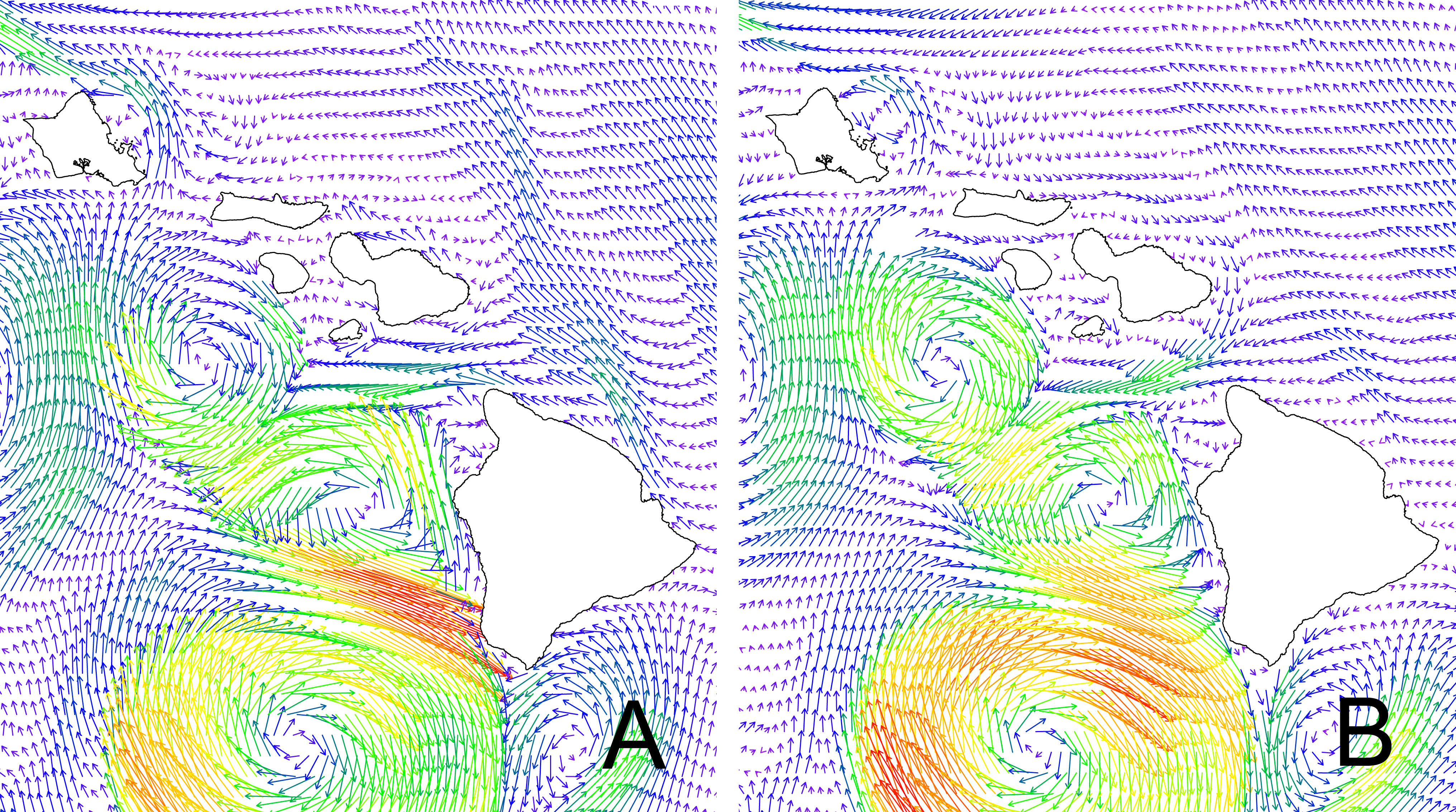

Supplement: Figure S7 — Differences in eddy pattern and strength in surface layers (A, 2.5 m) vs. deep layers (B, 55 m) on March 31, 2011. Arrowhead direction follows current direction, and u/v velocity is displayed through arrow length and color (purple, low velocity, red, high velocity). While large gyres remain consistent at different depths, smaller features vary along this gradient. For example, the currents around Kaho‘olawe, the small gyre off the eastern coast of O‘ahu, and currents to the north of Maui all vary in direction and/or velocity. [file peerj-06-5688-s007.png]
